# Invasive Pulmonary Aspergillosis in Critically Ill Patients with Hantavirus Infection, Austria

**DOI:** 10.3201/eid3006.231720

**Published:** 2024-06

**Authors:** Stefan Hatzl, Laura Scholz, Florian Posch, Philipp Eller, Alexander C. Reisinger, Martin Zacharias, Gregor Gorkiewicz, Martin Hoenigl, Ines Zollner-Schwetz, Robert Krause

**Affiliations:** Medical University of Graz, Graz, Austria (S. Hatzl,^,^ L. Scholz, F. Posch, P. Eller, A.C. Reisinger, M. Zacharias, G. Gorkiewicz, M. Hoenig, I. Zollner-Schwetz, R. Krause);; BioTechMed-Graz, Graz (S. Hatzl, G. Gorkiewicz, M. Hoenigl, R. Krause)

**Keywords:** hantavirus, viruses, invasive aspergillosis, intensive care unit, critical illness, Austria, fungi

## Abstract

We investigated a cohort of 370 patients in Austria with hantavirus infections (7.8% ICU admission rate) and detected 2 cases (cumulative incidence 7%) of invasive pulmonary aspergillosis; 1 patient died. Hantavirus-associated pulmonary aspergillosis may complicate the course of critically ill patients who have hemorrhagic fever with renal syndrome.

Hantaviruses are a large group of rodentborne single-stranded RNA viruses that cause clinical illness of varying severity in humans. Austria, and especially its southernmost federal states (Styria, Carinthia, and Burgenland), are endemic regions for the hantavirus puumala virus (PUUV), as well as for Dobrava/Belgrade virus (DOBV) in rare cases (*1*). Both hantavirus entities cause hemorrhagic fever with renal syndrome (HFRS); case-fatality rates of 0.1%–1% in PUUV patients and up to 12% in DOBV patients have been reported (*2*). HFRS is characterized by strong inflammation affecting vascular endothelial cells, leading to thrombocytopenia and potentially to disseminated intravascular coagulopathy. HFRS leads to renal failure of varying severity; half of patients develop respiratory symptoms. Hypoxia might lead to intensive care unit (ICU) admission for patients who need oxygen supply or organ support (*3*–*6*).

Invasive pulmonary aspergillosis (IPA) has been increasingly reported as a serious and potentially lethal complication in patients who require ICU treatment for severe influenza or COVID-19–associated acute respiratory failure. Although exact ICU admission rates and treatment characteristics (e.g., mechanical ventilation, hemodynamic shock) in the context of HFRS are lacking, hantaviruses have been shown to cause direct damage to the airway epithelium, potentially enabling aspergillus to invade tissue (*7*). We therefore speculated that there is a risk for invasive pulmonary aspergillosis (IPA) in critically ill patients with HFRS.

## The Study

We performed a retrospective observational study, enrolling all consecutive adult patients with HFRS admitted to the ICU Medical University of Graz in Graz, Austria, during 2003–2023, and determined the rate of IPA. We screened all patients with clinical suspicion of HFRS and detection of PUUV or Hantaan virus (HTNV)/DOBV IgM by immunoassay (Reagena, https://www.reagena.com) (Appendix). All patient data were uniformly collected as described previously (*1*,*8*). The institutional review board of the Medical University of Graz approved the study (approval no. 33-329 ex 20/21), which we conducted in accordance with the Declaration of Helsinki (Version Fortaleza, 2013).

We classified the patients according to the European Confederation of Medical Mycology/International Society for Human & Animal Mycology consensus criteria for COVID-19–associated invasive pulmonary aspergillosis and influenza-associated pulmonary aspergillosis (*9*) by 2 infectious disease specialists who were blinded against the baseline characteristics. We performed all analyses using Stata version 16.1 (StataCorp., https://www.stata.com) and R version 4.0.5 (https://www.r-project.org). We reported continuous data as medians with 25th–75th percentiles; we summarized categorical data using absolute frequencies and percentages. We estimated median survival of the overall cohort by the reverse Kaplan–Meier method. We analyzed risks for development of hantavirus-associated pulmonary aspergillosis (HAPA) and death from any cause using competing risk cumulative incidence estimators.

Of 370 patients with HFRS, 29 (7.8%) were admitted to the ICU and therefore included in our study; median follow-up time was 4.3 years (Table 1). A total of 28/29 (96%) had PUUV-caused HFRS; 1 patient had DOBV-caused HFRS, confirmed by PCR. A total of 17/29 (59%) patients reported contact with rodents or rodent excreta. All patients had fever, 21/29 (72%) patients had headache and 13/29 (45%) had diarrhea. At ICU admission, the median sequential organ failure assessment (SOFA) score was 10 (7–15) and the median paO_2_/FiO_2_ ratio (Horowitz index) was 150 mm Hg (97–188 mm Hg). Almost all patients (27/29 [92%]) received oxygen supply upon ICU admission. A total of 19/29 (66%) patients received noninvasive ventilation support (Table 1). The remaining 9 patients received invasive mechanical ventilation; 3 received additional extracorporeal membrane oxygenation (ECMO). Hemodialysis was necessary in 19/29 (66%) patients. A total of 5 patients died during intensive care treatment, corresponding to a 10-day ICU survival of 86.2% (95% CI 67.3–94.5), 30-day ICU survival of 86.2% (67.3–94.5), and 90-day ICU survival of 82.7% (63.4%–92.4%). No patients died outside the ICU (Table 1; Appendix).

**Table 1:** Baseline characteristics of 29 hantavirus patients hospitalized in ICU, Graz, Austria*

Characteristic	Value
Age, y (range)	51 (44–65)
Sex	
F	5 (17)
M	24 (83)
BMI, kg/m^2^	26.2 (24.1–28.1)
Known contact with rodents or rodent excreta	17 (59)
Concurrent conditions	
Hypertension	8 (27)
Diabetes mellitus	1 (4)
Atrial fibrillation	2 (8)
Thromboembolic disease	4 (13)
COPD	2 (7)
Asthma	2 (8)
Prior cancer in remission	1 (34)
Signs and symptoms at diagnosis	
Headache	21 (72)
Eye pain	2 (7)
Fever >38.5°C (101.3°F)	29 (100)
Body temperature, °C (range)	39.5 (39.2–40.1)
Diarrhea	13 (45)
Abdominal pain	11 (38)
Kidney pain	3 (10)
Blurred vision	2 (8)
Time from first symptom to diagnosis, d	3 (1–4)
Median blood counts at diagnosis (range)	
Leukocytes, G/L	10.2 (6.9−14.7)
Neutrophils, G/L	8.2 (5.5−12.5)
Lymphocytes, G/L	0.7 (0.6–0.9)
Hemoglobin, g/dL	15.2 (11.4–17.5)
Platelets, G/L	58 (33–74)
Median laboratory results at diagnosis (range)	
Creatinine, mg/dL	4.2 (1.6–6.6)
eGFR, mL/min	16.3 (8.0–40.9)
Urea, mg/dL	117 (69−178)
C-reactive protein, mg/L	131 (95−176)
PCT, mg/L	8.5 (2.6−19.5)
Interleukin-6, pg/mL	151 (56–410)
Bilirubin, mg/dL	0.59 (0.5–0.8)
AST, U/L	46 (30–99)
ALT, U/L	46 (26–135)
LDH, U/L	390 (271–696)
Median coagulation test results (range)	
Prothrombin time, %	90 (65–98)
aPTT, s	41.0 (36.1–49.1)
D-Dimer, mg/L	11.5 (3.8−15.9)
ICU characteristics	
SOFA	10 (range 7–15)
paO_2_/FiO_2_ ratio	150 (range 97–188)
Oxygen supply	
None	2 (8)
Mask	7 (25)
HFNC	2 (8)
NIV-CPAP	10 (29)
Mandatory ventilation	9 (31)
vvECMO	2 (8)
vaECMO	1 (4)
Need for dialysis/hemofiltration	19 (66)
Need for vasopressors	14 (48)
Low-dose	10 (72)
High-dose	4 (28)
Outcomes	
Proportion of HAPA	2 (6.9)
Deceased	5 (17)
Length of ICU stay, d (range)	4.5 (2.8−10.3)
Length of hospital stay, d (range)	13.0 (9.0–28.0)

*Values are no. (%) except as indicated. ALT, alanine aminotransferase; aPTT, activated prothrombin time; AST, aspartate aminotransferase; BMI, body mass index; COPD, chronic obstructive pulmonary disease; eGFR, estimated glomerular filtration rate; HFNC, high-flow nasal cannula; ICU, intensive care unit; LDH, lactate dehydrogenase; NIV-CPAP, noninvasive ventilation continuous positive airway pressure; SOFA, sequential organ failure assessment; vaECMO, venoarterial extracorporeal membrane oxygenation; vvECMO veno-venous extracorporeal membrane oxygenation.

We observed 2 cases of probable HAPA 2 days and 7 days after ICU admission with a total proportion of 6.9% (95% binomial exact CI 0.1–22.8) of patients during the ICU stay. One patient had PUUV and the other had DOBV infection. Their laboratory findings included positive bronchoalveolar lavage galactomannan >1.0 optical density index (2/2 patients), bronchoalveolar lavage culture growing *Aspergillus* species (2/2), and positive serum GM optical density >0.5 ODI (1/2), in addition to the other required parameters defining HAPA. Neither patient exhibited any notable concurrent conditions before ICU admission. Both patients received prednisolone, initiated after the first diagnostic test indicating pulmonary aspergillosis; daily dose was 1 mg/kg body weight for supportive treatment of acute respiratory failure (Table 2). The cumulative incidence of HAPA was 3.4% (95% CI 0.3–14.9) at 5 days, 7% (1.2–20.1) at 10 days, and 7% (1.2–20.1) at 90 days after ICU admission. After diagnosis of HAPA, we observed a 10-day ICU survival of 100%, 30-day survival of 100%, and 90-day survival of 50% (0.6%–91%) (Appendix).

**Table 2:** Characteristics of 2 patients with hantavirus pulmonary aspergillosis and mycological criteria, Graz, Austria*

Characteristic	Patient 1, DOBV infection	Patient 2, PUUV infection
EORTC risk factors	None	None
APACHE II score	36	26
Thoracic CT scan/radiograph	Bilateral dense infiltrates	Bilateral dense infiltrates
Steroids to treat pneumonia	Yes	Yes
Renal replacement therapy	Yes	Yes
Vasopressor	High-dose nordarenalin and vasopressin	High-dose nordarenalin and vasopressin
BAL *Aspergillus* culture	+	+
BAL *Aspergillus* qPCR	−	−
BAL galactomannan index	7,11	7,71
Serum *Aspergillus* qPCR	NA	NA
β-D-glucan, pg/mL	126	626
Serum galactomannan index	0.79	0.32
Mycologic criteria	4	3
Antifungal therapy	Voriconazole	Posaconazole
Outcome	Dead	Alive

*The table summarizes diagnostic and therapeutic characteristics of invasive pulmonary aspergillosis associated with hantavirus-disease. APACHE score, Acute Physiology and Chronic Health Evaluation score; BAL, bronchoalveolar lavage; CT, computed tomography; EORTC, European Organisation for Research and Treatment of Cancer; NA, not available; qPCR, quantitative PCR; +, positive; −, negative.

## Conclusions

We report invasive pulmonary aspergillosis as an infectious complication in critically ill patients with HFRS, without any classical risk factors for IPA. The 2 cases of HAPA observed in our study correspond to a cumulative incidence of invasive pulmonary aspergillosis of 7% in our ICU cohort, comprising 29 patients with HFRS. Of note, the corrected cumulative incidence rate including only patients with diagnostic tests for mold infections was 22.2% (95% CI 2.8%–60%).

During the COVID-19 pandemic and previous epidemic waves of influenza, secondary pulmonary mold infections and especially aspergillosis gained increasing attention (*9*–*11*). Previous studies investigating noninfluenza/non-COVID respiratory virus–associated invasive pulmonary aspergillosis nearly exclusively reported these co-infections in patients with well-known classical risk factors for IPA, mostly immunocompromised solid organ or hematopoietic stem cell transplant recipients (*12*–*14*). Those findings are in contrast with our findings for HFRS, in which neither of the 2 HAPA patients had underlying diseases predisposing to IPA or received immunosuppression before ICU admission. This fact is a major characteristic of HFRS patients who acquire their infections primarily during outdoor recreational activities or physical work in mouse-infested areas, thereby excluding almost all severely comorbid or immunocompromised persons (*2*). Therefore, we hypothesize that hantaviruses such as PUUV or DOBV may change the lung immune composition by viral pathogenic factors in a similar extent as COVID-19 or influenza to pave the way for *Aspergillus* infections.

The 2 patients of our cohort showed biomarkers indicative for IPA immediately after ICU admission. Therefore, if our findings are confirmed in larger studies, preemptive antifungal treatment or prophylaxis after ICU admission could be considered in intubated and mechanically ventilated hantavirus-infected patients. That possibility holds especially true for patients with acute respiratory failure requiring ECMO treatment or receiving corticosteroids, which both contribute to the risk for invasive mold infection (*11*,*15*).

This study’s limitations include its retrospective design and nonstandardized investigation of respiratory samples. The incidence of HAPA might have been higher than reported here if we included only patients with more severe HFRS or if fungal biomarkers were incorporated into diagnoses in all cases. The incidence rates of invasive aspergillosis may also depend on environmental factors. Therefore, our results may probably differ from observations in other centers. Finally, the relatively small number of critically ill patients included here limited our ability to examine risk factors for HAPA; prospective multicenter studies are warranted. In the meantime, clinicians should remain aware that HAPA may complicate the course of illness in HFRS patients.

Appendix Additional information about invasive pulmonary aspergillosis in critically ill patients with hantavirus infection.
