# Supplementary material for: Invasive Pulmonary Aspergillosis in Critically Ill Patients with Hantavirus Infection, Austria
Source: Emerg Infect Dis. 2024 Jun;30(6):1275–8. doi: 10.3201/eid3006.231720 (PMC11138969; doi:10.3201/eid3006.231720)
Supplement: Appendix — Additional information about invasive pulmonary aspergillosis in critically ill patients with hantavirus infection. [file 23-1720-Techapp-s1.pdf]

Article DOI: http://doi.org/10.3201/eid3006.231720

EID cannot ensure accessibility for supplementary materials supplied by authors. Readers who have difficulty accessing supplementary content should contact the authors for assistance.

Invasive Pulmonary Aspergillosis in Critically Ill Patients with Hantavirus Infection, Austria

Appendix

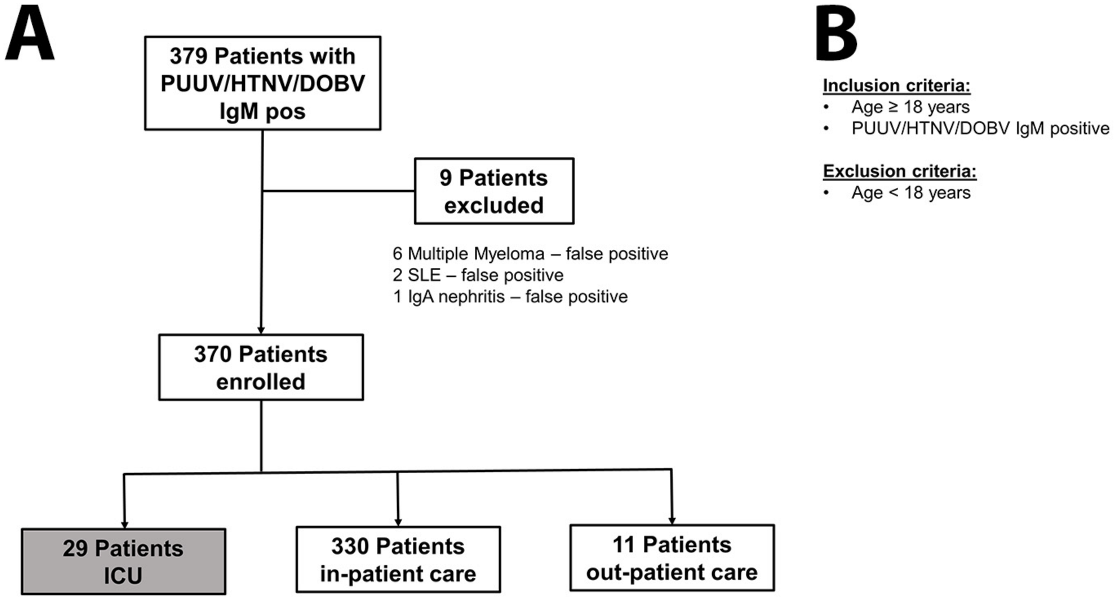

Appendix Figure 1. A) Full trial protocol and flow diagram of study of invasive pulmonary aspergillosis in critically ill patients with hantavirus infection. A total of 379 patients were initially enrolled. Nine patients were excluded due to false positive antibody testing and alternative diagnosis (6 multiple myeloma, 2 systemic lupus erythematosus, 1 IgA nephritis). Therefore, 370 patients were included in the analyses, whereof 29 were treated in the ICU. B) Inclusion and exclusion criteria of the study. PUUV, puumalavirus; HTNV, hantaanvirus; DOBV, dobrava-belgrade-virus; SLE, systemic lupus erythematosus; IgA, immunoglobulin A.

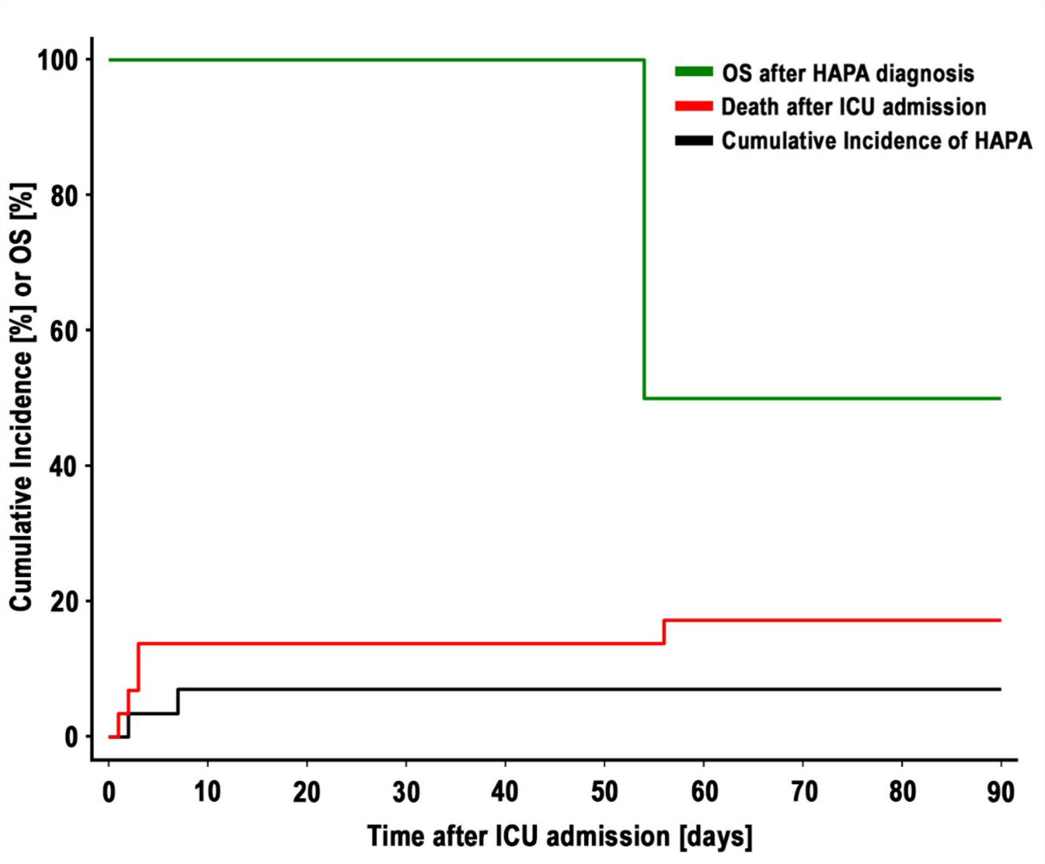

Appendix Figure 2. Cumulative incidence of HAPA and overall survival. Kaplan Maier and cumulative incidence estimators showing the primary endpoint of the study. The green line depicts the overall survival after HAPA diagnosis, the red line depicts mortality of the cohort after ICU admission and the black line depicts the cumulative incidence of HAPA in the cohort comprising 29 patients with death of any-cause as competing event. OS, overall survival; ICU, intensive care unit; HAPA, hantavirus associated pulmonary aspergillosis.
